# 

**Published:** 1884-11

**Authors:** 


### CONTENTS:

**Art. I.—Proceedings of British Dental Association.................................289**

**Art. II—Influence of Micro-Organisms in the Prodoction of Caries. By Arthur Underwood, L. D. S., M. R. C. S..................................307**

**Art. III—Transposition of Certain Functions of the Teeth. By J. Edw. Line. D D. S., M. D..................................................317**

**Art. IV.—Mucoid or Puruloid Engorgement of the Antrum. By John S. Smith, D. D. S..................................................323**

### EDITORIAL, ETC.

**The Dental Department of the University of Maryland..............................327**

**Dr. A. J. Volck's Elaine and Repousse Work......................................328**

### MONTHLY SUMMARY.

**Anatomical Reasons for Dento-Alveolar Abscess of the Hard Palate...............330**

**The Treatment of Carbuncle.......................................................334**

**Anæthesia per Rectum.............................................................335**

